# *Phaleria macrocarpa* Boerl. (*Thymelaeaceae*) Leaves Increase SR-BI Expression and Reduce Cholesterol Levels in Rats Fed a High Cholesterol Diet

**DOI:** 10.3390/molecules20034410

**Published:** 2015-03-09

**Authors:** Yosie Andriani, Tengku Sifzizul Tengku-Muhammad, Habsah Mohamad, Jasnizat Saidin, Desy Fitrya Syamsumir, Guat-Siew Chew, Mohd Effendy Abdul Wahid

**Affiliations:** 1 Institute of Marine Biotechnology, Universiti Malaysia Terengganu (UMT), Kuala Terengganu 21030, Terengganu, Malaysia; E-Mails: sifzizul@umt.edu.my (T.S.T.-M.); habsah@umt.edu.my (H.M.); ijaxzt@umt.edu.my (J.S.); desy@umt.edu.my (D.F.S.); effendy@umt.edu.my (M.E.A.W.); 2 Chemistry Department, Faculty of Mathematics and Natural Sciences, Universitas Bengkulu (UNIB), Bengkulu 38371, Indonesia; 3 School of Applied and Biomedical Sciences, Faculty of Science and Technology, Federation University Australia, Ballarat, 3350 Victoria, Australia; E-Mail: h.chew@ballarat.edu.au

**Keywords:** *Phaleria macrocarpa* Boerl, hypercholesterolemia, HDL, SR-BI

## Abstract

*In vitro* and *in vivo* studies of the activity of *Phaleria macrocarpa* Boerl (*Thymelaeaceae*) leaves against the therapeutic target for hypercholesterolemia were done using the HDL receptor (SR-BI) and hypercholesterolemia-induced Sprague Dawley rats. The *in vitro* study showed that the active fraction (CF6) obtained from the ethyl acetate extract (EMD) and its component 2',6',4-trihydroxy-4'-methoxybenzophenone increased the SR-BI expression by 95% and 60%, respectively. The *in vivo* study has proven the effect of EMD at 0.5 g/kgbw dosage in reducing the total cholesterol level by 224.9% and increasing the HDL cholesterol level by 157% compared to the cholesterol group. In the toxicity study, serum glutamate oxalate transaminase (SGOT) and serum glutamate pyruvate transaminase (SGPT) activity were observed to be at normal levels. The liver histology also proved no toxicity and abnormalities in any of the treatment groups, so it can be categorized as non-toxic to the rat liver. The findings taken together show that *P. macrocarpa* leaves are safe and suitable as an alternative control and prevention treatment for hypercholesterolemia in Sprague Dawley rats.

## 1. Introduction

Hypercholesterolemia is defined as a condition where the cholesterol level in human blood is higher than 200 mg/dL. These high cholesterol levels can be caused by an increase in triglycerides and LDL-cholesterol, which will cause the total cholesterol (TC) level to increase [1]. Hypercholesterolemia can be a causative agent for the development of atherosclerosis. The obvious way of treating the various forms of hypercholesterolemia is to try and reduce the blood cholesterol levels. High-density lipoprotein (HDL) particles play a critical role in cholesterol metabolism [2]. HDL particles are bound by hepatic scavenger receptor class B type I (SR-BI) present on the surface of liver cells. SR-BI mediates the selective transport of lipid from HDL to cells [3]. It was well established that HDL plays an important role in reverse cholesterol transport (RCT) by removing plasma cholesterol ester (CE) [4]. The CE-bound HDL is then transported to the liver where CE is up taken into the liver cells via SR-BI allowing the unbound HDL to carry out its function to further reduce the amount of plasma CE thus reducing the risk of atherosclerosis. Therefore, the role of SR-BI is crucial in order to allow HDL to remain functional to transport the plasma cholesterol away from the blood vessels to the liver to be metabolized [2,5,6]. SR-BI provides a new potential target for conventional drug treatment for atherosclerosis and also hypercholesterolemia.

Interest in the discovery of new chemical entities or drugs is greater than ever, due to the emergence of new diseases, the increasing number of drug-resistant diseases and pathogens, and, more importantly, the problem of finding new replacements and alternatives to current drugs that have adverse side effects on human health. Plants are a major source in the search for new drugs, especially plants with good ethnobotanical records, due to the advantages of the plants themselves, which can include low toxicity, abundance, low cost and lesser side effects if used in the right dose. Mahkota Dewa (*P. macrocarpa* Boerl., family Thymelaeaceae) has been used traditionally in Indonesia for the treatment of cancer and also to treat many diseases of the liver and heart, diabetes, skin diseases, rheumatism, as an anti-histamine, and to lower cholesterol levels (hypocholesterolemia). Some research regarding the usefulness of *P. macrocarpa* fruit and leaves has been published. Its advantages were attributed to the presence of alkaloids, polyphenolics, saponins, phalerin, and mangiferin [7]. Previous studies have reported the anticancer activity of *P. macrocarpa* leaves against HeLa [8] and myeloma cells [9]. Its fruits were also found to be active against HeLa cells [8], leukemia cells L1210 [10], and MCF-7 breast cancer [11]. Some synthesized benzophenone glucoside *P. macrocarpa* fruit derivatives showed anticancer activity against esophageal, stomach and prostate cancer cells [12], while its bark showed anticancer activity against L1210 cells [13]. Furthermore, the fruit extract was also found to possess anti-diabetic [14,15] and anti-inflammatory effects [16]. Ali *et al.* suggested that an isolated compound, mangiferin, may be responsible for the anti-hyperglycemic activity through extra-pancreatic action [17]. The antihypercholesterolemic activity of *P. macrocarpa* fruit extract in white rats was already reported [15,18]. The mechanism of action of this activity is thought to occur through enhancement of LDL receptor PCSK9 expression [19]. However, there is no research related to the ability of its leaves to reduce cholesterol levels (hypocholesterolemia) and the mechanism of action of this activity remains uninvestigated, therefore an *in vitro* study was done to evaluate the mechanism of action of *P. macrocarpa* leaves extract on reducing cholesterol levels (hypocholesterolemia) by looking at the expression of SR-BI. The *in vivo* study was continued to prove the hypocholesterolemia effect of *P. macrocarpa* leaves in hypercholesterolemia-induced Sprague Dawley rats. Moreover, the SGOT/SGPT activity and histology profiles of the liver were also explored in this study to investigate the toxicity of the extract.

## 2. Results and Discussion

### 2.1. Cytotoxicity Property

Prior to determining the ability of *P. macrocarpa* leaves and their components to increase SR-BI promoter activity and subsequently maybe identify potential agents for hypercholesterolemia and atherosclerosis, their cytotoxicity was examined first. This step is important to ensure that the extracts and compounds used to screen for the SR-BI promoter activity are not cytotoxic against the human liver HepG2 cell line. The cytotoxicity of extracts obtained by successive solvent extraction indicated by the IC_50_ value against the HepG2 cell line was less than 30 µg/mL (data not shown), indicating that none of samples should be considered cytotoxic and have potential for further investigation in the SR-BI promoter expression assay. Subsequently, the ethyl acetate extract (EMD) was chosen for further fractionation and compound isolation since the EMD extract yielded the highest SR-BI expression compared to the other extracts.

### 2.2. Fractionation, Compound Isolation, and Structure Elucidation of the Active Compound Isolated from P. macrocarpa Leaves

**Figure 1:**
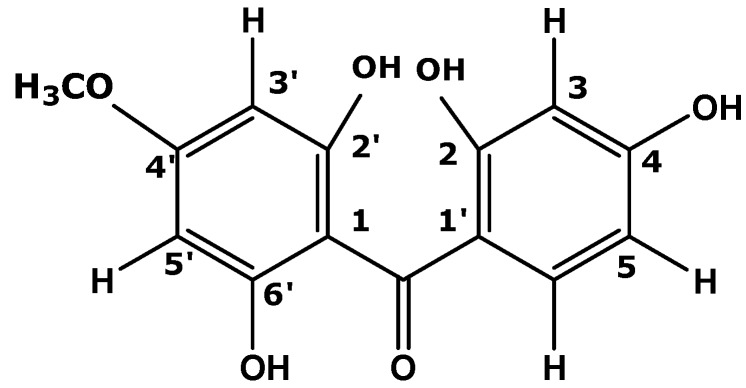
Molecular structure of 2',6',4-trihydroxy-4'-methoxybenzophenone.

The EMD extract was subjected to open column gravity chromatography eluting with a sequential gradient of hexane, chloroform and ethyl acetate. Fractions were collected, evaporated and checked by TLC using 4:6 CHCl_3_-ethyl acetate as solvent system. The fractions showing the same TLC pattern were combined, resulting in 10 fractions labelled CF1‒CF10. Fraction CF6 was recrystallized using CHCl_3_ to yield an active compound (340 mg, 22.67%). The compound was a yellow powder with a melting point of 130 °C. It gave a positive result for the ferric chloride reaction, revealing its phenolic nature. Its purity was checked by TLC [6:4 (*v*/*v*) chloroform-ethyl acetate, visualization was carried out by using UV-254 nm, iodine vapour, reagent spray with DPPH and vanillin] to show one spot with an R_f_ value of 0.65. The isolated compound was subjected to spectroscopic measurements and comparison of the results with literature data. Based on this it was determined to be a benzophenone. The GC-MS spectrum analysis suggested the molecular formula C_14_H_12_O_5_, indicated by the molecular ion peak at *m*/*z* 260. An [M−H]^+^ base peak was observed at *m*/*z* 259. The ^1^H-NMR spectrum showed the presence of an aromatic proton group, with signals at δ: 6.061 (s), and deshielded doublet signals centered at δ 7.650 and δ 6.881 (both 2H, *J* = 8.8 Hz), indicative of a *para*-substituted symmetric phenol ring, all of which agreed the notion of a benzophenone structure with hydroxyl (-OH) group substituents at carbons 2', 4 and 6'. The ^1^H-NMR spectrum also showed a methoxy group singlet at 3.819 (^13^C: δ 54.88). This position was assigned to C-4 by an HMBC correlation experiment. A signal at δ 196.94, typical for the carbonyl group of a benzophenone confirmed the structure hypothesis. The proton and carbon data and corresponding assignments are shown in Table 1 Data comparison with published data confirmed it to be the known benzophenone 2',6',4-trihydroxy-4'-methoxybenzophenone (Figure 1), previously isolated from fruit of *P. macrocarpa* [20].

**Table 1:** 1D-NMR (^1^H- and ^13^C-NMR) assignments (400 MHz, acetone-*d*_6_) for compound (C) compared to the literature.

Compound C from leaves of *P. macrocarpa*			
Position -C	^13^C-NMR (δ, ppm)	Position -H	^1^H-NMR (δ, ppm, *J* = Hz)
-C=O	196.94	-	-
C-2'	165.27	-	-
C-6'	161.62	-	-
C-4	161.13	-	-
C-1	132.21	-	-
C-3	131.60	H-3	7.650 (*d*, 8.8)
C-5		H-5	
C-2	114.36	H-2	6.887 (*d*, 8.8)
C-6		H-6	
C-1'	105.58	-	-
C-3'	93.64	H-3'	6.061 (s)
C-5'		H-5'	
C-4'OCH_3_	54.88	H-4'	3.819 (s)
		-OH	9.854

Other related compounds were isolated from the pit of Mahkota Dewa, namely 2,4,6-trihydroxy-4-methoxybenzophenone-2-*O*-*β-*d-glucoside (known as mahkoside A), together with mangiferin, kaempferol-3-*O-β-*d-glucoside, dodecanoic acid, palmitic acid and ethyl stearate [21]. Mangiferin A and mangiferin B (2,4,6-trihydroxy-4-methoxy-6-acetylbenzophenone-2-*O-β-*d-glucoside) have been isolated from the nut shell part of Mahkota Dewa [12].

### 2.3. In Vitro Study: Mechanism Evaluation of P. macrocarpa Leaves Extract in Reducing Cholesterol Level by Looking the Expression of SR-B1 Genes

**Figure 2:**
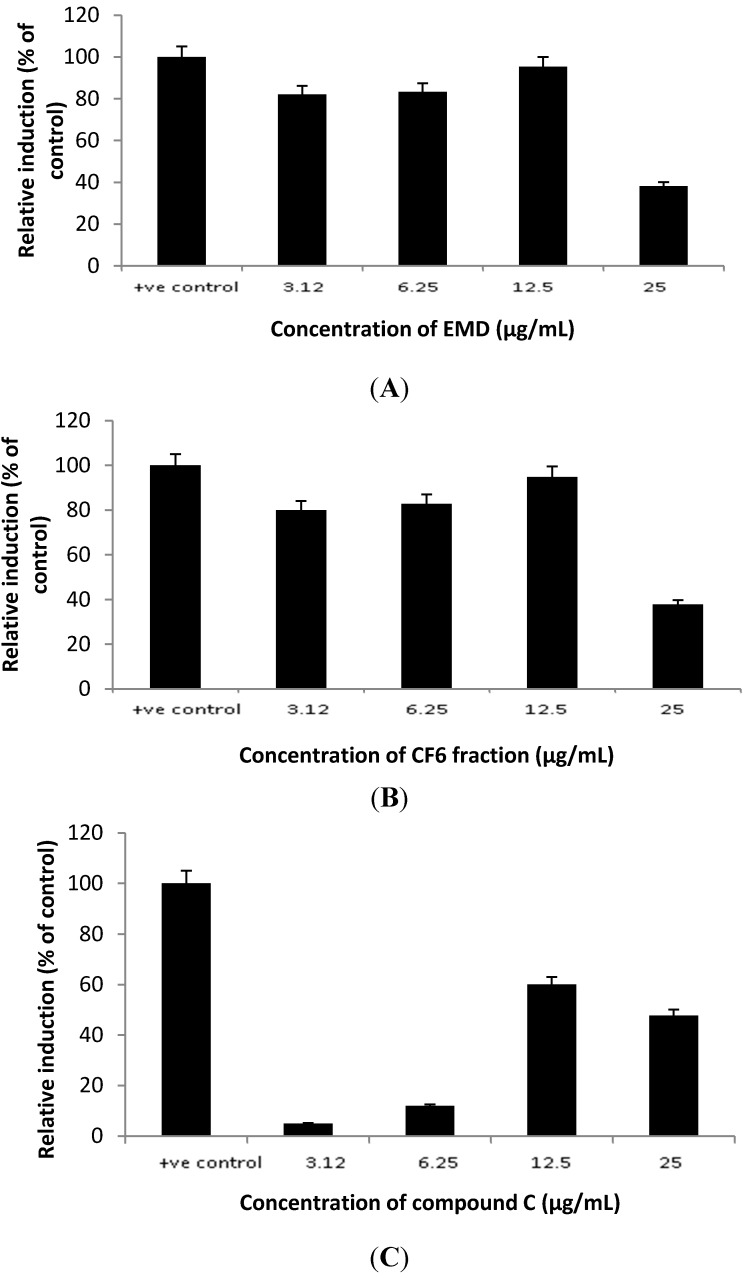
The effects of EMD (**A**), CF6 (**B**) and 2',6',4 trihydroxy-4' methoxybenzophenone (**C**) from *P. macrocarpa* leaves on the transcriptional activity of SR-BI promoter.

The active fraction of *P. macrocarpa* leaves (CF6) obtained from EMD and the compound 2',6',4-trihydroxy-4'-methoxybenzophenone isolated from CF6 increased SR-BI expression compared to the positive control rosiglitazone at 12.5 µg/mL (Figure 2). EMD and CF6 produced higher transcriptional activity of the SR-BI promoter as compared to the untreated samples. In all cases, 12.5 µg/mL produced the highest response, with both EMD and CF6 increasing the promoter activity of SR-BI up to 95% of the control (rosiglitazone), followed by the component C at 60% of control.

The isolated compound was thus found to show lower activity than its respective parent fractions, which indicated that there was a synergistic effect of the various constituents present in the fractions to increase the SR-BI promoter activity. This also indicated that the mechanism of action of *P. macrocarpa* leaves extract in reducing cholesterol levels might occur by increasing the expression of the SR-BI promoter.

SR-BI mediates the selective uptake of cholesterol esters from HDL [22,23,24]. Up-regulation of SR-BI in the liver may be antiatherogenic by increasing the catabolism of lipid from HDL and atherogenic lipoprotein [22]. It was well established that HDL plays an important role in reverse cholesterol transport (RCT) by removing plasma cholesteryl ester (CE) as well as accumulated CE along the lining of blood vessels to the liver, thus, reducing the risk of atherosclerosis. However, SR-BI not only mediates HDL-CE uptake, but also stimulates free cholesterol efflux from cells into HDL, suggesting that SR-BI in the vessel wall might play a role in cholesterol removal [25]. Other researchers have suggested that hepatic over-expression of SR-BI is associated with decreased plasma levels of HDL cholesterol transport into the bile [26,27,28]. Therefore, samples that can enhance SR-BI expression will probably have an effect in reducing cholesterol levels. Two other compounds (4',6-dihydroxy-4-methoxybenzophenone-2-*O-β-*d-gentiobioside or 4',6-dihydroxy-4-methoxybenzophenone-2-*O*-[*β*-d-glucopyranosyl-(1→6)-*β*-d-glucopyranoside] and 4',6-dihydroxy-4-methoxybenzophenone-2-*O-β-*d-glucoside, which were isolated from a methanol extract of *P. macrocarpa* leaves, were also reported to have activity in reducing cholesterol levels and act as anti-atherosclerosis agents by increasing the SR-BI expression [29].

### 2.4. In Vivo Study: Hypocholesterolemia Effect of P. macrocarpa Leaves in Hypercholesterolemia-Induced Sprague Dawley Rats

#### 2.4.1. Total Cholesterol Levels

**Figure 3:**
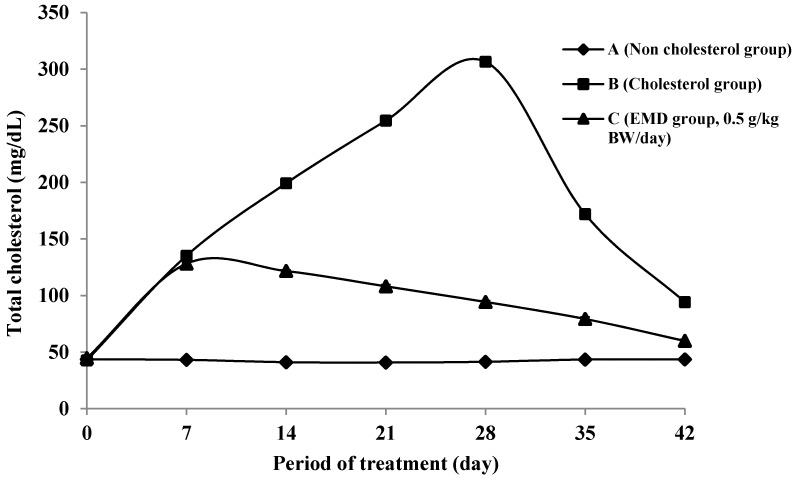
The changes of total cholesterol level during treatment on groups of non-cholesterol (A) (♦); cholesterol (B) (■); and EMD 0.5 g/kgbw(C) (▲).

Figure 3 shows the changes of total cholesterol level in rats during treatment. The average cholesterol level on day 0, classified as normal conditions, was 43.394 ± 1.552 mg/dL. This value is consistent with Malole and Pramono’s statement that the normal cholesterol concentration of rat blood is around 40–130 mg/dL [30]. After one week of administration of cholesterol-enriched food diet accompanied by propylthiouracil (PTU) to group B, the cholesterol level increased by as much as at 216% compared to baseline, thus it can be assumed that the rats had achieved hypercholesterolemia conditions. In the B group, hypercholesterolemia conditions were maintained until the 28th day of treatment.

The purpose of administration of PTU and cholesterol in the food pellets is to induce the rats to achieve hypercholesterolemia or hyperlipidemia conditions [31]. According to Grundy, a high cholesterol diet can increase blood cholesterol levels [32]. The addition of 2% cholesterol in the standard food for three days is able to cause hyperlipidemia in experimental rats [33]. PTU is an anti-thyroid substance that can impede the formation of thyroid hormone which has a role in lipolysis, so the obstruction of thyroid function will increase the concentration of blood cholesterol by increasing the biosynthesis of endogenous cholesterol receptor [34]. Moreover, the addition of fat or cooking oil can cause increase blood cholesterol levels as they contain many saturated fatty acids. According to Grundy [32], a diet of saturated fatty acids can suppress the LDL receptor activity, but the exact mechanism is still unknown.

The influence of the given extract in decreasing the total cholesterol level in group C is clearly seen on the 28th day of experiments after two weeks of treatment, with a 224.9% decrease in cholesterol level compared to the cholesterol group (B). This cholesterol level decrease in group C is very significantly different from group B (*p* < 0.05). Hence, the extract of EMD at 0.5 g/kgbw was proved to be able to reducing cholesterol levels in the tested rats. The hypercholesterolemia reduction from the 7th day to the 28th day of treatment showed that the EMD extract of *P. macrocarpa* leaves can be used as a preventive step against hypercholesterolemia. During washout time (after 28 days to 42 days), the administration of cholesterol-enriched food was stopped and substituted by standard (non-added cholesterol) food only. A further decrease in cholesterol level was observed in group C during this time.

#### 2.4.2. The HDL Cholesterol Level

**Figure 4:**
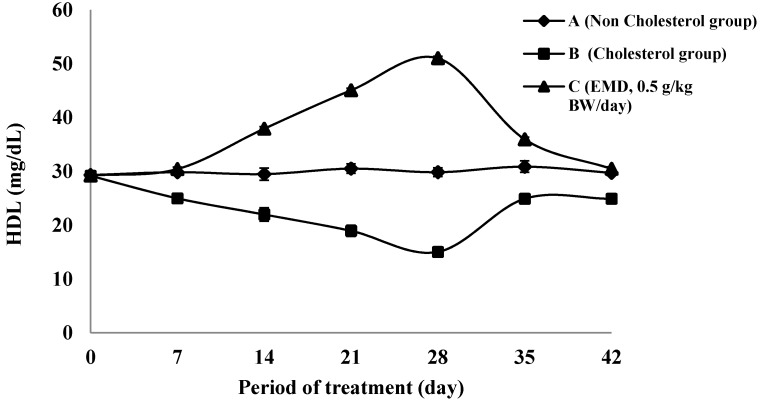
The changes of HDL-cholesterol level during treatment on groups of non-cholesterol (A) (♦); cholesterol (B) (■); and EMD 0.5 g/kgbw (C) (▲).

The changes in HDL cholesterol levels during the treatment step and washout are shown in Figure 4. The highest increase is obtained after the 28 days of treatment phase. It can be seen that the C group exhibited an increase in the percentage of HDL level by 239% compared to the cholesterol group (B). The HDL level of C group is significantly different compared to group B (*p* < 0.05). Meanwhile, the decrease of HLD level during the treatment period of B group was 94% compared to the baseline time value. The amount of increase in HDL level is related to the decrease of total cholesterol level in the EMD group. The phenomenon of decrease might be due to the occurrence of increasing HDL levels through a mechanism correlated with the enhancement of SR-BI gene expression in the liver.

The enhancement of SR-BI genes expression will increase the synthesis of HDL receptor in the liver, which can cause the HDL levels of the *in vivo* study animals to increase too. Studies in rodents suggest that SR-BI expression in the liver is very important for HDL metabolism and for overall *in vivo* cholesterol homeostasis [35]. A series of *in vitro* and *in vivo* studies of the activity, expression, and regulation of SR-BI provided strong indirect evidence of its relevance for HDL metabolism. Hepatic over- expression of SR-BI is associated with decreased plasma levels of HDL cholesterol transport into the bile [27,28,29], increased HDL cholesteryl ester clearance [27,36] and increased biliary cholesterol content and transport of cholesterol from the liver into the bile [26,37,38]. Meanwhile, SR-BI may protect against atherosclerosis by mediating the hepatic uptake and biliary secretion of HDL cholesterol and therefore may suppress atherosclerosis by stimulating and overall reverse cholesterol transport; and it may thus prevent accumulation of atherogenic lipoproteins in plasma [35].

#### 2.4.3. SGOT and SGPT Activity

In general, enzymes are excellent markers of tissue damage, as organ or tissue damage causes the release of increased amounts of many enzymes in the bloodstream [39]. Examples are SGOT and SGPT, which are used to detect the presence of liver damage. The measurement of SGOT was used to assess the toxicity of *P. macrocarpa* leaves extract on the liver. According to Girindra [40], the SGOT activity of rats in normal conditions is in the 45.7–80.8 U/I range. Other studies have variously reported that the normal SGOT in rat is around 124.80 ± 23.71 U/I [41], 105.5 ± 2.8 U/I [42], and 167.33 ± 13.27 U/I [38]. From this study, the average of normal SGOT activity obtained at the baseline time is in the 131.73–134.65 U/I range (Table 1) which still within the normal range according to the literature [41,43]. The influence of extract administration began to be seen from the 7th day until the end of treatment and washout. Group C exhibited SGOT activities ranging from 131.94–136.51 U/I which were not significantly different from those of group A until the end of treatment and washout (*p* > 0.05). This shows that the administration of EMD extract at 0.5 g/kgbw dosage was still safe for use and did not cause any disturbance to the rats’ liver functions. SGOT activity of 242.1 ± 36.4 U/I is considered to indicate toxicity and the ability to disrupt the liver function [44]. Thus, the results from this study showed EMD extract of *P. macrocarpa* leaves at the dose of 0.5 g/kgbw were not toxic to the liver with regard to SGOT activity. Cholesterol-enriched diets can cause toxicity in the liver of rats if more than 3% cholesterol is used [44]. Hepatic damage induced by cholesterol diet in rats, mice and rabbit has been noted often [27,45,46]. This proved that the hypercholesterolemia treatment in this research still falls in non-toxic category, in which the cholesterol was used at 1% dosage.

Changes of SGPT value are also a parameter used to assess the toxicity effects on the liver of the usage of EMD extract of *P. macrocarpa* leaves during the treatment. This enzyme appears after SGOT as the indicator of liver damage [47]. The range of normal SGPT in rat is 55.28–66.06 U/I [38], 53.25 ± 1.71 U/I [41], 128.17 ± 5.09 U/I [42], and ranged between 30–60 U/I [48]. At the baseline time, normal SGPT activity obtained in this study was in the range 64.46–64.87 U/I (Table 2).

**Table 2:** The SGOT and SGPT Activities of rat plasma during treatment on groups of non-cholesterol (**A**); cholesterol (**B**); and EMD 0.5 g/kgbw (**C**).

**Group**	**Activity of SGOT (U/I) for every 7 days during Treatment**						
	**0**	**7**	**14**	**21**	**28**	**35**	**42**
**(A)**	131.73 ± 0.89	133.71 ± 1.32	133.45 ± 0.93	134.20 ± 0.95	133.86 ± 0.85	133.18 ± 1.26	133.62 ± 1.05
**(B)**	134.32 ± 1.14	146.44 ± 0.77 *	157.84 ± 1.12 *	192.73 ± 0.59 *	236.73 ± 0.94 *	179.18 ± 0.68 *	145.51 ± 0.80 *
**(C)**	134.65 ± 0.68	133.69 ± 0.97	133.10 ± 1.46	133.50 ± 0.96	136.51 ± 0.91	131.94 ± 1.20	132.50 ± 1.63
**Activity of SGPT (U/I) at every 7 days during Treatment**							
**Group**	**0**	**7**	**14**	**21**	**28**	**35**	**42**
**(A)**	64.46 ± 1.19	63.95 ± 1.61	64.73 ± 1.23	64.96 ± 1.18	64.85 ± 1.12	65.15 ± 0.94	64.34 ± 0.53
**(B)**	64.66 ± 0.77	97.32 ± 1.66 *	138.25 ± 0.71 *	175.53 ± 0.63 *	222.991 ± 0.76 *	130.57 ± 1.22 *	75.57 ± 2.43
**(C)**	64.87 ± 0.86	65.98 ± 0.76	66.46 ± 0.94	64.95 ± 1.31	65.04 ± 1.28	64.87 ± 0.89	64.87 ± 0.96

Notes: * *p* < 0.05 significantly difference in comparison between cholesterol group, normal and treatment group; each value is presented as mean ± SD (*n* = 10).

The influences of the extract administration were observed on the 7th day of treatment time to the end of the treatment time (28th day) and through washout (42th day). The C group exhibited SGPT activity which is not significantly different from that of group A until the end of the treatment and washout (*p* > 0.05). The values obtained were in the 63.95–65.98 U/I range (Table 2). In other studies SGPT values were considered to be toxic and cause the damage to the liver function if in the 232.65 ± 17.38 U/I to 283.3 ± 30.1 U/I range [42,43]. Thus, it was concluded that EMD extract from *P. macrocarpa* leaves at 0.5 g/kgbw dosage did not cause any disturbance to the function of the experimental rats’ livers and it can therefore be categorized as non-toxic and safe to be used in a treatment.

#### 2.4.4. Histological Analysis

**Figure 5:**
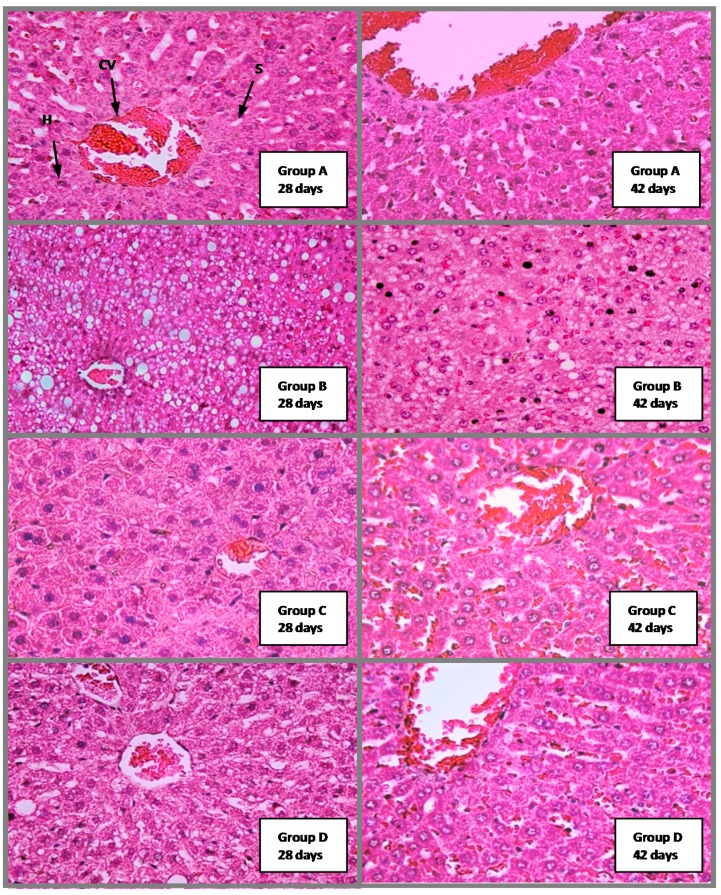
The Effect of *P. macrocarpa* leaves on liver of hypercholesterolemia induced rat of group A, B, C and D at 28 and 42 days. A: control non cholesterol group; B: control cholesterol group; C: 0.5 g/kgbw of EMD treated rats; D: 0.25 g/kgbw of EMD treated rats. CV = Central Vein; H = Hepatocytes; S = Sinusoids. H&E staining, magnification = 200×.

Figure 5 shows there was no toxicity effects or abnormalities in the liver tissue samples of group C on days 28 and 42 of treatment. Central vein, hepatocytes, and sinusoids showed a normal appearance and no fatty changes were seen, which indicates no metabolic disruption and no evidence of lesions as compared to normal rat’s liver tissue. Meanwhile, group B (the cholesterol group) showed many mild to moderate fatty liver symptoms at day 28, and showed mild fatty liver condition at day 42., which indicates the effect of giving cholesterol-enriched food to this group.

There were no evidence of fibrinous material on the endothelium lining in groups C and D at day 28, and histological analysis showed normal appearance of central vein, hepatocytes, and sinusoids, which indicates the absence of pathological conditions in these groups compared to the normal control group (A) at days 28 and 42. In group D at the 28th day, fatty liver was still substantial, but the amount was lesser comparatively the group B. This is probably caused by the EMD extract administration at 0.25 g/kgbw (50% dosage) which was not enough to reduce the cholesterol levels optimally in the liver of group D, as compared to group C at 0.5 g/kgbw (100% dosage). The decrease of fatty liver in the C group on the 28th and 42nd days compared to the cholesterol control group (group B) shows that the consumption of EMD extract can be used as a preventative step as well as a control step for hypercholesterolemia. Meanwhile, the absence of lesions and liver changes in this group showed that the EMD extract at 100% dosage was not toxic to the rat’s liver.

### 2.5. Proposed Mechanism of P. macrocarpa Leaves Effect in Reducing Cholesterol Levels (Hypocholesterolemia)

**Figure 6:**
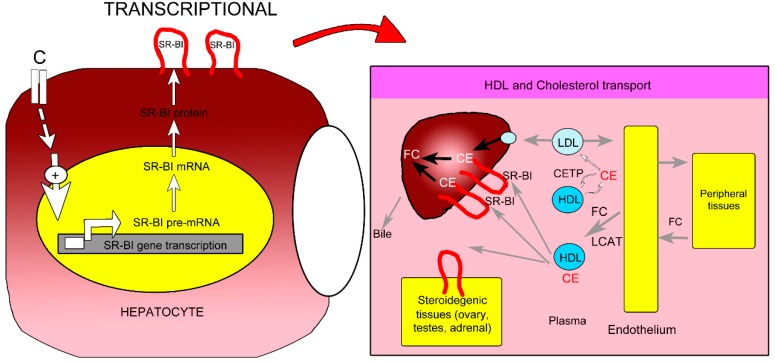
Proposed mechanism for hypocholesterolemia activity of compounds C from *P. macrocarpa* leaves through the enhancement of SR-BI gene expression at the transcription level (modified from Leiva *et al.* [49] and Krieger [50]).

Various studies have reported that an increase in SR-BI gene expression led to an increase in the amount of SR-BI present on the surface of liver cells [26,27,28]. Therefore, the increment of SR-BI production at the transcription level by compound C increased the uptake of HDL-CE bound to the SR-BI on the hepatocytes. CE were then hydrolyzed to free the bound cholesterol, resulting in increased FC release. The FC in hepatocytes are mostly metabolized to bile acids or are eliminated directly through the bile or feces. This may reduce the total cholesterol levels which might be the mechanism of action of *P. macrocarpa* leaves for reducing cholesterol levels or has a hypocholesterolemic effect (Figure 6).

The plasma cholesterol levels in cholesterol metabolism are regulated by exogenous and endogenous pathways [49]. The exogenous pathway shows cholesterol from dietary and biliary sources is absorbed in the intestine and ultimately enters the circulation as a component of chylomicrons, whereas the intestine is the primary site of the exogenous pathway of dietary cholesterol uptake. In the endogenous pathway, cholesterol is synthesized by the liver and extra hepatic tissues, and enters the circulation as a component of lipoproteins into plasma, or is secreted into bile. There are two options available to regulate plasma cholesterol: block its uptake into the body with agents such as selective cholesterol absorption inhibitors, or promote its removal from the circulation by blocking the endogenous pathway with agents such as statins [51]. However, other endogenous pathway may be used to regulate plasma cholesterol levels, such as through regulation of SR-BI. The SR-BI is most abundantly expressed in the liver, and the expression of SR-BI in hepatocytes is critical in controlling plasma HDL-cholesterol levels. Because SR-BI is indeed directly involved in the regulation of hepatic HDL-cholesterol uptake and biliary excretion of HDL-cholesterol, the presence of this receptor in the liver is closely related to the control of the levels of HDL-cholesterol in plasma as well as the overall rate of reverse cholesterol transport [13,35,36,50].

## 3. Experimental Section

### 3.1. P. macrocarpa Leave Materials

Samples of *P. macrocarpa* leaves were obtained from Bengkulu, Sumatera Island, Indonesia and were collected between July and September 2008. After collection, they were oven dried (temperature not exceeding 40 °C), and then they were ground to powder.

### 3.2. Extraction and Fractionations of P. Macrocarpa Leaves

The extraction of 500 g dried powder of leaves was carried out with following solvents successively: hexane, chloroform, ethyl acetate and methanol (adapted from Andriani *et al.* [52]). Each filtrate was evaporated under reduced pressure to yield the corresponding crude extract. Ethyl acetate crude extract (EMD; yield 2.5%) was selected for further fractionation since the EMD increased the SR-BI promoter expression during the bioassay. In this study, the fractionation of the actives crude extract was performed by silica gel gravity column chromatography, utilizing Merck Kiesel gel 60 PF_254_ Art No. 7734 of particle size 0.063–0.200 mm (Merck, Darmstadt, Germany) yielding a mixture of compound fractions. Further fractionations were carried out by silica gel gravity column chromatography using Merck Kiesel gel 60 PF_254_ Art No. 9385 of particle size 0.040–0.063 mm.

### 3.3. Isolation and Structure Elucidation

The EMD fraction (15 g) was subjected to open column gravity chromatography (30 cm × 4 cm) on Si gel 60 230–400 mesh, Merck). The column was packed by hexane and then followed by gradient elution of sequential hexane, chloroform and ethyl acetate. The fractions (200 mL each) were collected, evaporated and monitored by TLC using CHCl_3_‒EtOAc (4:6) as a solvent system. The fractions showing same TLC pattern were combined, resulting in 10 fractions namely CF1-CF10. Fraction CF6 was selected for further purification as it showed the highest SR-BI promoter expression increasing activity. Recrystallization of the active fraction was done using CHCl_3_ to yield the active compound C (340 mg, 22.67%), as a yellow powder with melting point 130 °C. The actives fraction (CF6) and pure compound (C) were subjected to *in vitro* study. Meanwhile, the actives crude extract (EMD) was subjected to *in vivo* study. The molecular formula of the isolated compound was determined on the basis of its NMR spectra (^1^H- and ^13^C-NMR) together with GC-MS. The NMR spectra were recorded using acetone-*d*_6_ on an Avance III 400 MHz. instrument (Bruker, Faellanden, Switzerland). GC-MS spectra were measured on a Thermo Finnigan (Bremen, Germany), trace GC instrument.

### 3.4. In Vitro Study: Mechanism Evaluation of P. macrocarpa Leaves in Reducing Cholesterol Level by Looking the Expression of SR-BI Genes

The HepG2 cell lines were obtained from the American Type Culture Collection (Rockville, MD, USA) and maintained in culture flasks (Corning, Oneonta, NY, USA) as described previously [43]. The HepG2 cells were kept in MEM media (Sigma, St. Louis, MO, USA) with 10% fetal bovine serum (ICN Biomedicals, Aurora, OH, USA) and 1% penicillin-streptomycin at 37 °C under 5% CO_2_.

#### 3.4.1. Cytotoxicity Study by MTT Assay

The 3-(4,5-dimethylthiazol-2-yl) 2,5-diphenyl tetrazoliumbromide (MTT) assay was used to determine the cell viability. This test reflects the cytotoxicity activity of *P. macrocarpa* leaves [53]. HepG2 cells were seeded at a density of 2.5 × 10^5^ cells/well on 96-well plates. Samples were prepared in various concentrations by serial dilution in MEM medium 30, 15, 7.5, 3.75, 1.875, 0.938 and 0.469 µg/mL. One hundred µL of sample was added into each well, then the plates were incubated for 72 h at 37 °C, 5% CO_2_. The assay of each concentration was performed in triplicates and untreated cell was used as control. After 72 h, 20 µL fresh MTT solution (5 mg/mL in PBS) was added to each well. The plate then was incubated for four hours at 37 °C, 5% CO_2_. Then, medium (170 µL) was removed from each well and DMSO (100 µL) was added and mixed thoroughly by pipetting 10–20 times. The plate was left for 30 min before reading by ELISA reader at 570 nm. Cytotoxic activity was expressed as fifty-percent inhibitory concentration (IC_50_), *i.e.*, the concentration that yields 50% inhibition of the treated cells compared to untreated cell control. The samples IC_50_ value of less than 30 µg/mL was considered not cytotoxic and had used for further investigation [54].

#### 3.4.2. Bioassay of *P. macrocarpa* Leaves against the Therapeutic Target for Hypercholesterolemia: SR-BI Promoter Trancriptional Activity Assay

pGL-3-SR-BI and pRL-CMV (Plasmid midi kit 100 protocol, cat no. 12145, Qiagen (Valencia, CA, USA) were prepared for inoculation.. One milliliter of pGL-3-SR-BI and pRL-CMV glycerol stock was transferred into 25 mL LB broth in a 50 mL Erlenmeyer flask, which was then stored in an incubator shaker at 37 °C for 16 h at 200 rpm. This was followed by centirifugation at 6000 rcf (≈8000 rpm) for 15 min, 4 °C. Afterward, the supernatant was removed, the pellet was taken and air dried. About 4 mL Buffer P1 was added and mixed into the pellet by vortexing. Four milliliters of P2 buffer were also added and mixed, followed by incubation at room temperature for 5 min. In the next step, 4 mL P3 buffer was added and the mixture was centrifuged at 12,000 rcf for 30 min, 4 °C. The supernatant was transferred to another backman tube (Kraemer Blvd, CA, USA) and re-centrifuged at 12,000 rcf for 15 min, 4 °C. The supernatant above was applied into the Qiagen-tip and allowed to enter the resin by gravity flow. The Qiagen-tip was washed by 2 × 10 mL QC. The eluate was collected in a 15 mL centrifuge tube. DNA was eluted with 5 mL QF by gravity flow. DNA was precipitated by adding 3.5 mL of isopropanol (100% *v*/*v*) mixing gently, and centrifuging immediately at 15,000 g for 10 min. The supernatant was carefully removed and the pellet air dried for 5–10 min. DNA was redissolved in a suitable volume (100–200 µL) of ddH_2_O. Then we proceeded to calibrate the concentration by Spectramax and double digestion of pGL3-SR-BI plasmid to confirm the inserts using XhoI and Hind III. Distilled water was use as a blank. Then electrophoresis was run to make sure the plasmid was present.

#### 3.4.3. Transfection of the Chimeric Reporter Plasmid into the HepG2 Cell Line

The SR-BI was sub-cloned into pGL3 luciferase reporter vector to increase expression and efficiency in transient transfection. In transient transfection, HepG2 cells were used (40–50 × 10^4^ cells). Then, cells were plated overnight if cells were confluence between 60%–80%. The next day cells were transfected with pGL3-SR-BI promoter fragment and pRL-CMV plasmid. In step 1 the desired amount of 40–50 × 10^4^ HepG2 cells were counted, plated onto 96 well plates, and incubated for 24 h. For transient transfection (step 2) about 600 µL of solution A (7.72 µL pGL3-SR-BI + 6.7 µL pRL-CMV + 585.68 µL SFM) was mixed with 600 µL solution B (24 µL lifofectin + 576 µL SFM) and the mixture was incubated for 10 min at room temperature, then transferred into a Petri dish (“transfected medium”); In the third step, medium was removed from each well (from step 1), the 96 wells plate was then washed twice with 5 mL PBS. Transfected medium (75 µL, from step 2) was aliquotted into the well, and incubated at 37 °C for 4–5 h.

#### 3.4.4. Treatment of the Transfected Cells with Sample

The dose response of rosiglitazone as positive control (0.04 µM) and sample in the range concentration 3.1; 6.3; 12.5; 2.0; 50.0; 100 µg/mL was determined. Sample was dropped into 96 wells plate (from step 3 above) and incubated for 24 h, 37 °C, then the luciferace activity assay was performed using a luminometer. Results are calculated as relative induction (the Percentage of the ratio of SR-BI promoter expression of a sample compared to the control).

### 3.5. In Vivo Study: Hypocholesterolemia Effect of P. macrocarpa Leaves in Hypercholesterolemia-Induced Sprague Dawley Rats

In the animal treatment study, 7 weeks old female 150–180 g Sprague Dawley rats, healthy and displaying normal activity, were used. Forty (40) animals were divided into four groups which consisted of ten animals per group. Group A (untreated group) served as negative control, and group B (cholesterol treated group) as positive control. Groups C and D were the treatment groups which were feed with cholesterol-enriched food and EMD extract at 0.5 g/kgbw and 0.25 g/kgbw, respectively. Treatment rats were treated once daily by oral administration using sterile ball-tipped gavages needles for 28 days, followed by another 14 days observation period (the washout or recovery period, time points of 35 and 42 days). They had a free access to water *adlibitum* during treatment.

Blood was withdrawn from the tail vein by using a sterile needle at days 0 (baseline), 7, 14, 21, 28, 35 and 42 to determine the cholesterol (total cholesterol and HDL), SGOT, and SGPT levels. During the washout period, none of the rats were treated with cholesterol-enriched food and they had free access to standard food pellets (10% of body weight) and water *adlibitum*. Cholesterol-enriched food was made by combining cholesterol 1%, vegetable oil 8.5%, PTU 0.5% and standard food until 100%. Every ingredient were mixed and made into pellets, which were then oven dried at 50 °C.

#### 3.5.1. Total Cholesterol Level (CHOD-PAP Method)

Ten microliters of blood serum and 1000 μL of reagent were put in a reactor tube. All the ingredients are mixed and incubated together at room temperature for 10 min. The ingredient was read at 500 nm wavelength using a Spectronic 20D spectrometer (Milton Roy Company, Warminster, PA, USA).About 1000 μL of reagent is used as a blank. The measurement of the cholesterol standard used 10 μL cholesterol and 1000 μL reagent. Cholesterol standard is serially diluted (0; 40; 80; 100; 120; 160; 200 mg/dL). A standard curve was plotted from the standard absorption results, and then the blood sample cholesterol was read off the standard curve [55].

#### 3.5.2. HDL Cholesterol Level

The HDL cholesterol level was analyzed enzymatically using kits for HDL cholesterol precipitant (Randox, Crumlin Co., Antrim, UK). The samples were undergoes precipitation process followed by CHOD-PAP assay as described by manufacture.

#### 3.5.3. SGOT and SGPT Activity

About 20–100 μL blood serum and 1000 μL reagent for SGOT were put in a reactor tube. All the ingredients are mixed and incubated together in a water bath at room temperature for one minute. The ingredient absorption was read at 365 nm wavelength using a Spectronic 20D instrument. Then, the data was calculated with the formula:

SGOT activity (I/U) = 3235 × (Δ_absorption_/3) × (100/volume of sample) (1)

About 20–100 μL blood serum and 1000 μL reagent for SGPT were put in a reactor tube. All the ingredients are mixed and incubated together in a water bath at room temperature for one minute. The ingredient absorption was read at 365 nm wavelength using a Spectronic 20D instrument. Then, the data was calculated with the formula:

SGPT activity (I/U) = 3235 × (Δ_absorption_/3) × (100/volume of sample) [56] (2)

#### 3.5.4. Histological Analysis

Each selected rat was anesthetized and euthanized according to the study needs. For the rats’ anesthesia, gauze was soaked in 20 mL of 20% diethyl ether and then placed in the euthanasia chamber. The chamber was left closed with its lid on for 5 min in order to let the euthanasia agent vaporize. The rat which needed to be anaesthetized was placed in the chamber for 10 min until it fell unconscious, with no whisker movement and no pain reaction when its leg palm was pressed. Then the rat was immediately used for other protocols such as blood collection. For euthanatization, the method used was the overdose inhalation of ether anesthetic method [57]. The rat which needed to be euthanized was placed in a desiccator chamber containing 30 mL of 20% diethyl ether for more than 20 min or until it died, observing such sign as eye colour turning from red into white, no heartbeat and no whisker movement. After the organs were collected, the samples were put through the fixation process in which the tissues were soaked in 10% buffered formalin for 48 h, and then the liver was cut at different lobes in cross-sections of no more than 10 mm thickness and immersed in the 10% buffered formalin before proceed to tissue processing using a Tissue Processor Machine (Leica, Nussloch, Germany) for 24 h. This process involved dehydration using a graded alcohol series (from 70% to 100%) clearing in xylene and impregnation. Dehydrated samples then were embedded in plastic cassettes with paraffin wax using an Embedding Machine (Leica, Nussloch, Germany). Before sectioning, the blocks were trimmed to remove excessive wax. All samples were sectioned into 4 μm in length by using a rotary microtome machine (Leica, Nussloch, Germany), then the tissue was floated in a water bath at 38 °C for 3 min. The floating sample was fished out with clean glass slides coated with Mayer’s albumin which acted as tissue adhesive. Slides were placed in a staining jar and deparaffinized by submerging into three series of absolute xylene for 4 min followed by 100%, 95%, 90%, and 70% ethanol for 4 min for each percentage. Next, slides were washed under running tap water for 2 min. Then, slides were submerged into Harris haematoxylin (Sigma-Aldrich, Steinberg, Germany) for 2 min and then washed under running tap water for 2 min. The slides were next submerged in 1% acid alcohol for three dips to decolorize them and washed under running tap water for 2 min. Next, slides were submerged into 2% potassium acetate for 3 min and again washed under running tap water for 2 min. After that, slides were submerged into eosin for 2 min followed by washing under running tap water for 2 min. Stained slides were dried for 24 h at 38 °C. Before observation, slides were dipped into absolute xylene for 1 min and finally mounted with cover slip using DPX mounting. The sections of liver from toxicity study were examined under a light microscope for toxicity evaluation and photomicrograph was taken by using image analyzer microscope (Leica, Nussloch, Germany) at 200× magnification.

### 3.6. Statistical Analysis

Analyses data were done in triplicates and expressed as mean ± SD. Statistical comparisons were performed by one way analysis of variance (ANOVA) followed by Turkey test using SPSS Version 16.0 software packages (SPSS^®^ Inc., Chicago, IL, USA). The results were considered statistically significant if the *p* values were 0.05 or less among groups.

## 4. Conclusions

Successive extraction of *P. macrocarpa* leaves yielded EMD as the most active extract. The compound obtained from EMD was identified as a known compound, 2',6',4-trihydroxy-4'-methoxybenzophenone, which showed a 60% increase in transcriptional activity of SR-BI promoter. The *in vivo* study proved the effect of EMD at 0.5 g/kgbw dosage in reducing total cholesterol levels by 224.9% and increasing the HDL cholesterol level by 157% compared to a cholesterol group after 28 days of treatment. The decreased total cholesterol phenomenon might be due to the occurrence of increasing HDL leveld through a mechanism correlated to the enhancement of SR-BI expression in the liver. The enhancement of SR-BI expression will increase the synthesis of HDL receptor in the liver, thus the HDL level of the *in vivo* study will be increased and total cholesterol will be reduced. Furthermore, the results of SGOT and SGPT assays showed that EMD extract at 500 mg/kg dosage did not cause any disturbance to the function of rat liver and it can therefore be categorized as non-toxic. The findings showed that *P. macrocarpa* leaves are safe and suitable as an alternative control and preventive agent for hypercholesterolemia in Sprague Dawley rats.
